# P311: A cross-sectional survey on the incidence of sharps injuries among healthcare workers at 26 hospitals in China

**DOI:** 10.1186/2047-2994-2-S1-P311

**Published:** 2013-06-20

**Authors:** S Xu

**Affiliations:** 1 Infection control department, West China hospital of Sichuan University, Chengdu, China

## Introduction

Sharp injuries (SIs) among healthcare workers (HCWs) are an under-recognized problem in China. Prevention of SIs is particularly challenging, in part due to a dearth of information regarding SIs incidence in China.

## Objectives

We conducted a cross-sectional survey in 26 hospitals in Sichuan province, southwest China, to understand the incidence and evaluate the burden of SIs among HCWs.

## Methods

Twenty-six hospitals (20,997 beds in total), 13 tertiary and 13 secondary hospitals, located in 12 municipal areas in Sichuan were selected for this cross-sectional survey after consulting the Bureau of Health of Sichuan. Subjects were HCWs who might have contacted sharps contaminated with blood and body fluids during the work. All subjects were surveyed for SIs that occurred on August 2011 using a baseline questionnaire. Those who reported SIs except injuries caused by ampoules or vials during the study period were interviewed face-to-face by infection control practitioners in each hospital.

## Results

A total of 22,573 out of 24,550 eligible HCWs (91.9%) were surveyed. There were 1,680 (7.4%) HCWs with 2,310 SIs that occurred during one month, corresponding to 11 SIs per 100 hospital bed in a single month. The highest incidence of SIs was seen in interns and nurse students (10.4%), followed by nurses (8%), doctors (7%), workers and housekeepers (4.2%) and technicians (3.9%). Unfortunately, only 3.9% HCWs reported their SIs, suggesting a very low reporting rate. The source patient could be traced in 70.6% of cases with SIs and 7.5% of SIs were caused by contacting with a source patient known carrying blood-borne pathogens (BBP). HBV (93 cases, 54%) was the most common BBP encountered, followed by *Treponema pallidum* (11%), HIV (4%), HCV (3%) and others (28%). Most SIs of nurses occurred in inpatient wards, while those of doctors were seen in operating rooms. SIs occurred in many procedures, particularly common when withdrawing intravenous kit, recapping needles, disposing medical waste, using surgical stitch, adding drugs to the liquid for intravenous and putting needles into sharps containers.

## Conclusion

SIs is a serious threat for HCWs in Sichuan, China, with a high incidence but an extremely low reporting rate. Occupational exposure to BBP among HCWs should be set up a priority to be tackled at both individual hospital and national level.

## Disclosure of interest

None declared

